# The development of prosodic focus marking in French

**DOI:** 10.3389/fpsyg.2024.1360308

**Published:** 2024-07-25

**Authors:** Emilie Destruel, Louise Lalande, Aoju Chen

**Affiliations:** ^1^Department of French & Italian, University of Iowa, Iowa, IA, United States; ^2^Institute for Language Sciences, Utrecht University, Utrecht, Netherlands

**Keywords:** focus, prosody, acquisition, children, French

## Abstract

**Introduction:**

French is traditionally described as a language favoring syntactic means to mark focus, yet recent research shows that prosody is also used. We examine how French-speaking children use prosody to realize narrow focus and contrastive focus in the absence of syntactic means, compared to adults.

**Method:**

We elicited SVO sentences using a virtual robot-mediated picture-matching task from monolingual French-speaking adults (*N* = 11), 4- to 5-year-olds (*N* = 12), and 7- to 8-year-olds (*N* = 15). These sentences were produced with narrow focus on either the subject or the object and contrastive focus on the object.

**Results:**

Linear mixed-effects logistic regression modeling on duration, mean intensity, mean pitch, and pitch range of the subject and object nouns showed that the 4- to 5-year-olds did not use any of these prosodic cues for focus marking but the 7- to 8-year-olds distinguished narrow focus from non-focus through an increase in duration, mean intensity and to a lesser degree, mean pitch in the object nouns, largely similar to the adults, and tended to use mean pitch for this purpose in the subject nouns, different from the adults, who used duration.

**Discussion:**

Our study corroborates previous findings that French-speaking 4- to 5-year-olds do not use prosody for focus. Further, it provides new evidence that 7- to 8-year-olds use prosody to mark narrow focus on the object in a more adult-like manner than narrow focus on the subject, arguably caused by a more dominant role of syntactic means in the subject position in French. Together, these findings show that syntax-dominance can influence both the route and the rate of acquisition of prosodic focus marking.

## 1 Introduction

The notion of *focus* plays a central role in communication by indicating to the hearer which part of the sentence is non-presupposed, and thus intended by the speaker to be asserted. Across languages, substantial variation is observed in the strategies used to signal focus. While some languages rely heavily on syntactic and lexical means, others primarily use prosodic means (Vallduvi and Engdahl, 1996; Kügler and Calhoun, 2020). French is commonly described as favoring syntactic strategies, especially in marking focus on grammatical subjects, where clefting seems to be most appropriate (e.g., Lambrecht, 1994; Féry, 2001). Nevertheless, many studies on French prosody show that information structure does exert an influence on prosody. Post-focus deaccentuation, prosodic phrasing, phonetic cues, like pitch height and duration, and choice of tonal patterns all play a role in encoding focus when syntactic means are not readily available (e.g., Féry, 2001; Chen and Destruel 2010; Beyssade et al., 2015; Lee, 2015). However, prosodic realization of focus in French-learning children has rarely been examined, in contrast to a wealth of research on children acquiring languages that primarily use prosody or use both prosody and word-order for focus-marking purposes (see Chen, 2018 for a review).

Our study seeks to address this gap by providing new empirical data on how French-speaking 4- to 8-year-olds use prosody to realize focus in French, compared to French-speaking adults. We are specifically interested in sentence-initial and final positions and two different types of focus, i.e., narrow vs. contrastive focus. Our study will further our understanding on how cross-linguistic differences in focus-marking shapes the rate and route of acquisition of prosodic focus-marking.

The remainder of the paper is structured as follows: We first briefly review literature on the realization of focus in French and French-speaking children’s use of prosody in comprehending and realizing focus (section 2). We present our research questions and hypotheses in section 3, and discuss our methodology in section 4. We report on our statistical analysis and results in section 5. Finally, section 6 concludes.

## 2 Background

### 2.1 Focus and its realization in French

In the literature on information structure, the notion of *focus* is commonly understood as the part of the sentence that makes available a set of alternatives that the speaker takes to be salient, and which in turn conveys information about how utterances fit in to the larger discourse structure (Rooth, 1992; Krifka, 2008). For example, when focus is marked on the grammatical subject *Alice* (1a), it indicates that the alternative propositions relevant for interpretation are of the form “*x bought candy*.” Similarly, when focus is marked on the grammatical object *a pie* (1b), it indicates that the hearer knows Alice bought something but does not know what was bought.

(1) a. [Alice]_F_ bought candy.
b. Alice bought [candy]_F_.

Moreover, scholars have commonly distinguished (at least) two types of focus depending on how the focal alternatives are exploited in the sentence (Kiss, 1998; Gussenhoven, 2007; Zimmermann and Onea, 2011). First, the term *information focus* is used to refer to information that introduces completely new information into a discourse, often diagnosed by identifying the open variable in a congruent wh-question (2a). Second, a *contrastive focus* is taken as an alternative to an expression that has previously been introduced into the common ground, and often expresses a correction to false assumptions (2b).

(2) a. Q: What did Alice buy? A: Alice bought candy (information focus).
b. Q: Did Alice buy popcorn? A: (No,) Alice bought candy (contrastive focus).

These two focus types can be accompanied by a variation in the scope of the focus domain: *broad focus* in (3a) refers to cases where entire syntactic constituents are focused (i.e., phrases, clauses, sentences), whereas *narrow focus* in (3b) correlates to a single element (i.e., nouns, verbs, adjectives) (e.g., Gussenhoven, 2007).

(3) a. Q: What happened? A: [Camille went to Paris]_F_ (broad focus).
b. Q: Who went to Paris? A: [Camille]_F_ went to Paris (narrow information focus).

While it is largely assumed that the focus element in a sentence is associated with prominence – compared to material that is already given in the discourse – the exact way to achieve this prominence may differ across and within languages (Kügler and Calhoun, 2020; Ladd and Arvaniti, 2023). To that end, West Germanic languages such as English and Dutch are known to primarily use prosodic means, i.e., placing a pitch accent on the focus element. Other languages such as Chadic languages rely on morphological markers. For example, Schwarz (2009) notes that in Buli, a morphosyntactic focus marker is required under the subject focus condition, which contains a particle either preceding or following the verb stem. This is illustrated in (4) below (from Schwarz, 2009: 952).

(4) Who ate the beans? ‘THE/ THIS WOMAN ate them.’
          nípōōwá lē ŋɔbī.
          woman.DEF PTL eat.

Complicating the matter further, a theoretical asymmetry between utterances with subject and object focus is also widely noted across different languages: the default position for prosodic prominence typically falls on the right-edge of a clause (i.e., in object position), leading subject focus sentences to be marked. French, the language of interest in this paper, illustrates this asymmetry well. Lambrecht (1994), among others, argues for a strong preference to use syntactic constructions like fronting and clefting (5) in the context of subject focus, while Destruel (2013) finds these are much less common in the context of object focus.

(5) a. Camille, elle est. allée à Paris. ‘*Camille, she went to Paris.*’ (fronting)
b. C’est. Camille qui est. allée à Paris. ‘*It is Camille who went to Paris.*’ (clefting).

Accordingly, past studies on French prosody have argued that information structure can affect prosody when syntactic means are not available. For example, some research shows that the focused element can be marked by a specific rising contour, which is both higher in pitch and aligned later than a typical final accent in non-contrastive contexts (*cf. accent d’insistance*, Di Cristo, 1996, or focus accent, Jun and Fougeron, 2000). Other studies also find that the prefocal region is characterized by a reduced pitch range and amplitude of tonal movements and by a reduced number of phrase boundaries (Touati, 1987; Jun and Fougeron, 2000; Dohen and Loevenbruck, 2004), and that the postfocal region can be characterized by an absence of prominent pitch movements (Rossi, 1985; Touati, 1987; Di Cristo and Hirst, 1993; Di Cristo, 1996; Clech-Darbon et al., 1999; Jun and Fougeron, 2000; Féry, 2001; Dohen and Loevenbruck, 2004). More recent studies demonstrate that the initial rise, which is an optional tonal Low-High unit associated with the left edge of the accentual phrase (or AP, the smallest unit of phrasing above the prosodic word level in French), can be an informative cue to focus in that it is more likely to occur at the left edge of a contrastive focus domain (D’Imperio et al., 2012; German and D’Imperio, 2016; Portes and Reyle, 2022). Yet, the clearest consensus across studies on focus and prosodic features in French is that prosodic phrasing, i.e., the grouping of words into phonological units of different sizes, is one of the main strategies employed by French speakers to mark the difference between given and new or contrastive elements. Specifically, a number of studies have reported a tendency for a narrow-focused constituent to be parsed in a separate AP (Féry, 2001; Dohen and Loevenbruck, 2004; Beyssade et al., 2009; Chen and Destruel, 2010; Michelas et al., 2014; Portes and Reyle, 2022). In operational terms, if two elements would typically be grouped into a single AP in a non-focused context, then in case one of them is focused, a prosodic restructuring process isolates that element in a separate AP.

In sum, French presents a great degree of variation due to prosody being only one of the options available to adult speakers in marking focus (Beyssade et al., 2009). This creates ambiguous input for children, which bears the question of how this might affect their acquisition. The next section turns to reviewing past literature on L1 acquisition of prosodic focus-marking in production across languages.

### 2.2 Acquisition of prosodic focus-marking in L1

Previous developmental research has generally suggested that although children can use prosody to distinguish new and given information to their interlocutor at the two-word stage in the second year of life, they do not acquire fully adult-like competence in production and comprehension of the prosody-to-information structure mapping until the age of 10 or 11 (Ito, 2018; Chen et al., 2020). Across languages, children acquire adult-like use of prosody in focus-marking in production at different rates and via different routes. Chen (2018) proposes that these differences are related to typological differences in prosodic focus-marking in the ambient languages, including reliance on phonetic means, transparency of form-function mappings, the use of the prosodic means in the lexical context and the importance of prosodic means in comparison to other means for focus marking. Specifically, children acquire the ability to use phonetic means, such as the phonetic implementation of phonological categories like lexical tones in Mandarin, lexical pitch accents in Swedish, and pitch accents in English, to distinguish narrow focus from non-focus, and differentiate different focus types at an earlier age in languages that exclusively rely on phonetic means for focus marking, like Mandarin (Yang and Chen, 2018). This happens compared to languages that use both phonological and phonetic means for focus marking, such as English (Hornby and Hass, 1970; Wonnacott and Watson, 2008), and Dutch (Chen, 2011; Romøren, 2016). Furthermore, children acquire phonological encoding of narrow focus earlier in languages with a more transparent form-function mapping between phonological means and focus conditions (e.g., Swedish vs. Dutch) (Romøren, 2016; Romøren and Chen, 2022). Transparency also affects phonological focus marking in different sentence positions within the same language. For example, Dutch-speaking children acquire phonological focus marking earlier in sentence-final position than in sentence-initial position where the phonological form-function mapping is less clear (Chen, 2011). Moreover, the timing of acquiring pitch-related cues for focus marking differs based on whether pitch is also used for lexical purposes (e.g., Mandarin vs. Dutch), with children acquiring the use of duration cues earlier than pitch-related cues in languages like Mandarin. Lastly, the relative importance of prosody and non-prosodic means such as word order for focus marking affects children’s use of phonetic means in distinguishing focus types in different syntactical settings. For example, 4- to 5-year-olds acquiring languages that use word order in conjunction with prosody to mark focus, like Finnish (Arnhold et al., 2016), use prosody more extensively and are less restricted by word order compared to children acquiring languages where prosody plays a primary role in focus marking, such as German (Sauermann et al., 2011) and Dutch (Chen and Höhler, 2018).

With respect to French, the literature on children’s acquisition of prosodic focus marking is very scarce compared to that on children acquiring a West Germanic language, and existing research has mainly examined perception and comprehension rather than children’s production skills. More specifically, Szendrői et al. (2017) examined the acquisition of the comprehension of prosodic focus marking in English-, German- and French-speaking children but has relevant implications for French-speaking children’s use of prosody in focus-marking in production and will be briefly reviewed here. The authors tested adults and children between the ages of 3 and 6 in their ability to recognize focal constituents in subject and object conditions through manipulations of prosody. All three languages allow the use of prosody in focus marking but differ in the use of syntactic means especially in subject focus (preferred in French, possible in German, dispreferred in English). In the experiment, the children were given visual stimuli in the form of three animal-tool pairs (subject-object, e.g., bird-hammer) and subsequently given false assumptions by the experimenter, where focus was marked on the target constituent depending on the condition. The children were expected to correct the target constituent (animal in subject condition and item in object condition) using the construction in (6), where the focal constituent is represented in italics.

(6) a. Experimenter: The bird has the *bottle*, right? Child: No, the *hammer*. (object focus condition)
b. Experimenter: The *bird* has the bottle, right? Child: No, the *hedgehog*. (subject focus condition)

Through this experiment, Szendrői et al. (2017) established that the English-, German- and French-speaking children could perceive focus in subject and object through prosodic cues and make corrections accordingly. However, they noted that the French-speaking children were more reluctant to give subject corrections, in comparison to the English-speaking children, suggesting that there are more natural ways to achieve this contrast in French, such as via clefting, and a possible difference in the acquisition of prosodic realization of focus in subjects and objects.

There is, to our knowledge, only two studies published on the production of focus by French-speaking children. First, Ménard et al. (2006) tested articulatory and acoustic correlates of contrastive focus in French-speaking 4- to 8-year-old children and adults, recording their repeated productions of the name ‘Baba’ in two carrier sentences as responses to the experimenter’s questions in neutral and contrastive focus conditions. Results showed that the children were not adult-like in using variations in intensity, formant and articulatory strategies (i.e., lip movements) to signal contrastive focus by the age of 8. Second, Esteve-Gibert et al. (2021) investigated the use of prosodic cues (i.e., syllable duration, word-level pitch range) and co-speech head gestures in the marking of focus types by French-speaking 4- to 5-year-olds, and the interaction between these two strategies. In their experiment, the participants were shown a visual display depicting a girl, Claire, with her eyes covered, and a bag containing one or more items. They were asked to interact with Claire by producing sentences containing noun phrases that would help her select a specific item in the bag indicated in the visual display (e.g., *prends [la moufle orange]*_NP_—‘take [the orange mitten]’_NP_). The trials with the bag containing just one item were used to elicit broad focus; the trials with the bag containing more than one item were used to elicit contrastive focus or corrective focus on either the noun or the colour adjective. Esteve-Gibert et al. (2021) found evidence for the use of gestures but no evidence for the use of syllable duration and pitch range expansion to distinguish focus types in the French-speaking 4- to 5-year-olds, different from their peers acquiring a language where prosody plays a larger role in focus marking, such as Finnish (Arnhold et al., 2016), German (Sauermann et al., 2011) and Dutch (Chen and Höhler, 2018).

## 3 Research questions and hypotheses

Given the theoretical and experimental backdrop introduced in the previous sections, and the scarcity of studies on production of focus in French-speaking children, our study sets out to examine the following two research questions using a phonetic approach:

- How do French-speaking children aged 4 to 8 years modulate prosodic parameters to signal narrow information focus (hereafter narrow focus) and narrow contrastive focus (hereafter contrastive focus), compared to French-speaking adults?
- Does the position of the focus constitute in the sentence (i.e., subject or sentence-initial vs. object or sentence-final) influence acquisition of prosodic focus marking?

Past studies on prosodic focus marking in French have been concerned with word- or syllable-duration (Lee, 2015; Esteve-Gibert et al., 2021), intensity (Lee, 2015), mean pitch (Lee, 2015), and pitch range (i.e., the difference between the highest and lowest pitch) (Esteve-Gibert et al., 2021; Portes and Reyle, 2022) in their phonetic analysis on the target words in different focus conditions. Following these studies, we focus on similar prosodic parameters at the word level, i.e., word duration, mean intensity, mean pitch, and pitch range.

Considering the findings from prior literature on acquisition of prosodic focus-marking and the role of prosodic and syntactic means in focus-marking in French, we hypothesize that French-speaking children will be unable to manipulate the above-mentioned prosodic parameters to indicate focus, regardless of focus type and sentence position, at the age of 4- to 5-years, but will develop certain aspects of this ability by the age of 7- to 8-years (**Hypothesis I**). Indeed, prior literature suggests that, in languages that allow for prosodic focus marking but recognize other (non-prosodic) strategies as preferential, these preferred strategies may precede the dispreferred ones in the development of the given language (Szendrői et al., 2017). Consequently, speakers of French and other languages that prefer alternative (non-prosodic) strategies, such as syntactic means, may acquire prosodic focus marking later than speakers of languages that favour prosodic means, such as English, Dutch, Mandarin and Swedish. Further, due to the preference for other non-prosodic strategies for focus marking in subjects in French and in the light of Szendrői et al.’s (2017) finding on the asymmetry in French-speaking children’s comprehension of focus in subjects and objects, we hypothesize that French-speaking children will have earlier acquisition of prosodic focus marking in objects than in subjects (**Hypothesis II**).

## 4 Methods

### 4.1 Participants

A total of 38 participants participated in this study. Of these, 11 were adult female speakers, 27 were children. All were monolingual native speakers of French, with normal hearing and vision. They all came from the Toulouse area in Southern France, and spoke French with no noticeable regional accent. The children were recruited from the Puygouzon primary school in the city of Albi. They consisted of two age groups: 12 were 4- to 5-year-olds and 17 7- to 8-year-olds. Details about their age range, average age and sex are provided in Table 1.

**Table 1:** Participants’ biographical details.

	Age range (years;months)	Average Age (years;months)	Sex
Children group 1 (*n* = 12)	4.7–5.11	5.1	5 females
Children group 2 (*n* = 15)	7.2–8.3	7.1	8 females
Adults (*n* = 11)	17–44	31.4	11 females

The two age groups were chosen for the following reasons. First, 4- to 5-year-olds are competent at producing multi-word sentences, which allowed us to study their use of prosody in focus marking in syntactically complete sentences. Second, previous studies on development in prosodic focus marking across languages (see Chen, 2018 for a review) have shown that children are adult-like in all or most of the aspects of prosodic focus marking at 7 or 8 years. Including 7- to 8-year-olds made it possible to study not only the developmental changes from 4- and 5-years to 7- and 8-years, but also find out whether French-speaking children become adult-like in prosodic focus marking at a similar age to children acquiring other languages. Finally, the eliciting task used in this study has been shown to be suitable for children aged 4 years and older. Using the same method to elicit data from two different age groups can facilitate the comparison in results between them.

### 4.2 Materials and procedure

The elicitation task in this study was adapted from the picture-matching game used by Chen (2011), which was also used in previous studies on children acquiring typologically different languages (Chen, 2018). It was designed to elicit SVO declarative sentences in the form of responses, where focus was marked in varying positions according to the target condition. Since the subject NP was always in sentence-initial position and the object NP in sentence-final position, we refer to the locus of focus as the position of focus and use the terms initial-position and final position interchangeably with subject and object in what follows. We tested three focus conditions: (i) narrow focus on the subject (NSF) which was triggered by who-questions (7a), (ii) narrow focus on the object (NOF), triggered by what-questions (7b), and finally (iii) contrastive focus on the object (NOCF), formulated as corrections to false assumptions (7c).

(7) a. Q: Qui mange les raisins? ‘*Who eats the grapes?*’
  A:  [Le lapin]_F_ mange les raisins. ‘*[The rabbit]*_F_ *eats the grapes.*’
b. Q: Que. découpe la mamie? ‘*What does the grandma cut?*’
  A:  La mamie découpe [le jambon] _F_ ‘*The grandma cuts [the ham]*_F_’.
c. Q:  Est-ce que le garçon peint une fleur? ‘*Does the boy paint a flower?*’
  A:  Non, le garçon peint [un ballon]_F_ ‘*No*, *the boy is painting [a ball]*_F_*’.*

**Figure 1:**
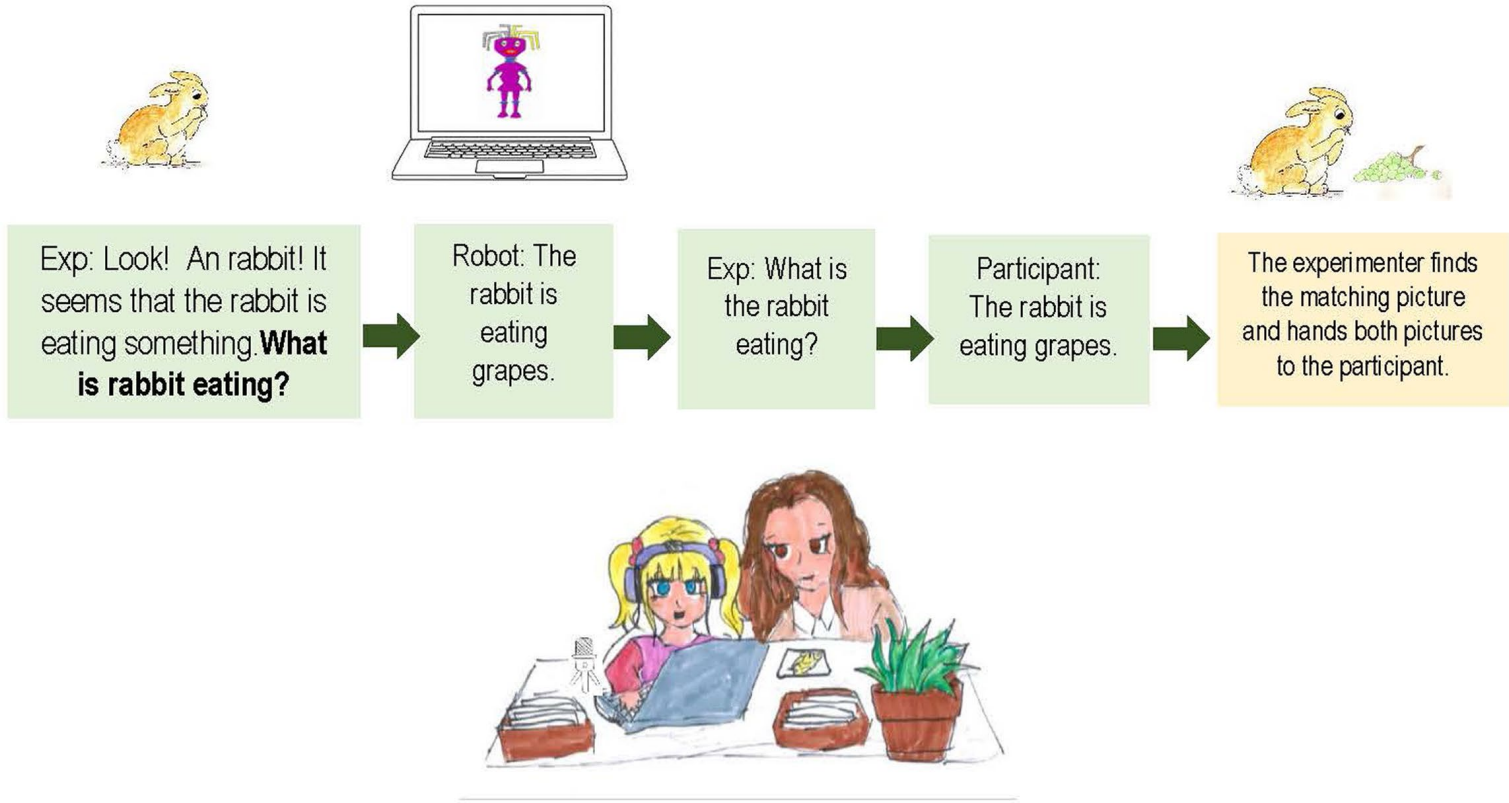
Description of the steps in the virtual robot-mediated picture-matching game.

The procedure took the form of a structured game, in which the participant and the experimenter sat in front of a computer screen and boxes of pictures together and the participant was supposed to help the experiment with finding the matching picture for each of her pictures. On each trial, the experimenter took an incomplete picture from a box (e.g., a rabbit that seems to be eating something). The experimenter then drew the participant’s attention to the picture and established what the picture was by stating “Look, a rabbit!” The experimenter then described what seemed to be missing in the picture and asked the congruent wh-question about the missing entity in the NSF and NOF conditions, which could be either the subject or the object (see Figure 1). In the NOCF condition, the experiment asked a yes-no question, making a guess about the missing object (e.g., *Is the rabbit eating a strawberry?*).

Upon hearing the question on each trial, the participant turned to a virtual robot assistant on the computer screen, which was there to help them get the correct answer. The participant clicked on the robot picture displayed on the screen and heard the answer in SVO form that included the missing part, via their headphones. The robot’s sentences were constructed from words recorded by a female native of French (age 35, speaker of standard Parisian French) in a randomized word list such that they contained no sentence-level prosody. Then, the experimenter repeated the question and the participant was instructed to respond to the question, using the exact same words used by the robot but speaking normally, instead of sounding like the robot.^1^ Finally, the experimenter looked for the picture of the missing part in the box and handed both pictures to the participant.

A total of 28 pairs of images were created, spread over 4 practice items, 8 NSF, 8 NOF and 8 NOCF. The subject and object nouns were all disyllabic words that were familiar to French-speaking 4- to 5-year-olds. These words were taken from French textbooks used in elementary schools. Where possible, words with sonorants and voiced consonants were used to facilitate annotation for pitch analysis. Each subject noun in the NSF condition occurred also in the NOF condition; each object noun occurred in all focus conditions. A full list of stimuli is provided in the Supplementary material (section 1.2).

The adults were tested individually in a quiet room by a female native speaker of French (first author) on the campus of the university of Albi, and were paid a small fee for their participation. The children were tested individually by a near-native French-speaking female research assistant on site in their classroom, but outside of class time. Each participant was randomly assigned to one of two semi-randomised stimulus orders. One stimulus order was the reversed order of the other. Each session began with a brief chat to help the participant become more comfortable with the experimenter. Following this, the experimenter explained the game and started the game with four practice trials. If a child provided elided or full-sentence answers but with pronouns or appeared to imitate the robot’s way of speaking during practice, the experimenter reminded him/her of the game rules. This was only needed for a small number of 4- to 5-year-olds. Most children responded with full sentences in their own prosody right from the start. Each test sessions lasted approximately 45 minutes for the younger children, about 35 minutes for the older children and less than 30 minutes for the adults. All sessions were audio-recorded using a portable digital recorder with a 48 kHz sampling rate, 16-bit resolution, and an external high-quality microphone positioned 10–15 cm from the participants. Nevertheless, the participants were not instructed to stay seated in the exact same position, so the distance between the microphone and the children’s mouth could not be precisely controlled for.

### 4.3 Data selection and annotation

The audio recordings from each participant were first orthographically annotated in Praat (Boersma and Weenink, 2022). During that first phase of the analysis, we identified three types of issues in children’s production, which we labeled rephrasing, fluency and experimenter errors. *Rephrasing* describes trials in which participants did not exactly answer the questions using the robot’s words, but instead used alternative focus strategies like clefting or fronting, inserted additional words (e.g. *non* ‘no’ preceding the answer in the contrastive condition), or gave a one-word answer. *Fluency* refers to trials in which participants hesitated (identifiable by long pauses), copied the robotic speech patterns, or used inadequate speech, such as speaking too rapidly or too softly. Finally, *experimenter error* refers to the trials in which the question was not repeated before a given response. The trials produced with one of these errors were removed from analysis.

Moreover, if the same errors were frequently produced by the same participant across stimulus items in a specific condition (or throughout all conditions), the data from that condition or that participant altogether was removed from analysis. This decision led to removing data from two participants in the younger group (4–5 years olds). Additionally, one of the items in the NOCF condition was eliminated in the analysis of all participants due to an issue in the formulation of the answer given the question asked.^2^ Thus, instead of 8 items, we report only 7 in the NOCF condition. The Supplementary material (section 1.1) gives an overview of the number of usable trials, per condition and group, that entered our analysis.

The target words (*n* = 1,113), i.e., the subject and object nouns, in the usable sentences (*n* = 575) were then annotated for word boundaries, following standard procedures (Machač and Skarnitzl, 2009) using Praat (Boersma and Weenink, 2022). Acoustic measurements including duration (ms), mean intensity (dB), mean pitch and pitch range (Hz) were subsequently extracted from these words using ProsodyPro (Xu, 2013). Finally, mean pitch and pitch range were checked for possible pitch-tracking related errors, such as halving- and doubling errors. In the case of 296 of the target nouns, mean pitch and pitch range were manually measured in Praat (Boersma and Weenink, 2022) and corrected in the data files.

## 5 Statistical analysis and results

We assessed the data using linear mixed-effects modeling in R Studio (RStudio Team, 2020) with the lme4 package (Bates et al., 2015). The outcome variables were duration, mean intensity,^3^ mean pitch and pitch range. The random factors were *Participant* and *Item*. Two sets of analyses were conducted for each outcome variable in the data in order to jointly address our research questions.

In the first set of analyses, we compared the nouns in the NSF and NOF conditions (where the subject noun was focused in the former but not in the latter, and vice versa for the object noun) to study the use of prosody in distinguishing narrow focus from non-focus, i.e., the effect of focus. The fixed factors included in the analyses were *Focus* (i.e., narrow focus or non-focus), *Position* (i.e., initial or final). In the second set of analyses, we examined the potential differences in the expression of contrastive focus and narrow focus, i.e., the effect of focus type. Since all target items were produced in object (final) position, the factor *Position* was no longer relevant. The trials used in this set of analyses were those in the NOF and NOCF conditions. The analyses only included *FocusType* (i.e., narrow focus or contrastive) as a fixed factor.

In both sets of analyses, to examine whether a specific prosodic cue was used to distinguish between two focus conditions, we built models using the aforementioned factors, in a stepwise fashion. Starting with a base model that only included the random factors (with no random slopes due to singular-fit issues), we then added the main effects of the fixed factor(s), and the two-way interaction between the two fixed factors (only in the first set of analyses). Model comparisons were conducted using the ANOVA function in RStudio, in order to derive the model with the best fit. We note that, for all results, we will only focus on statistically significant results concerning the fixed factors *Focus* and *Focus type* and their interaction with another fixed factor.

### 5.1 The effect of focus: narrow focus vs. non-focus^4^

#### 5.1.1 Adults

**Figure 2:**
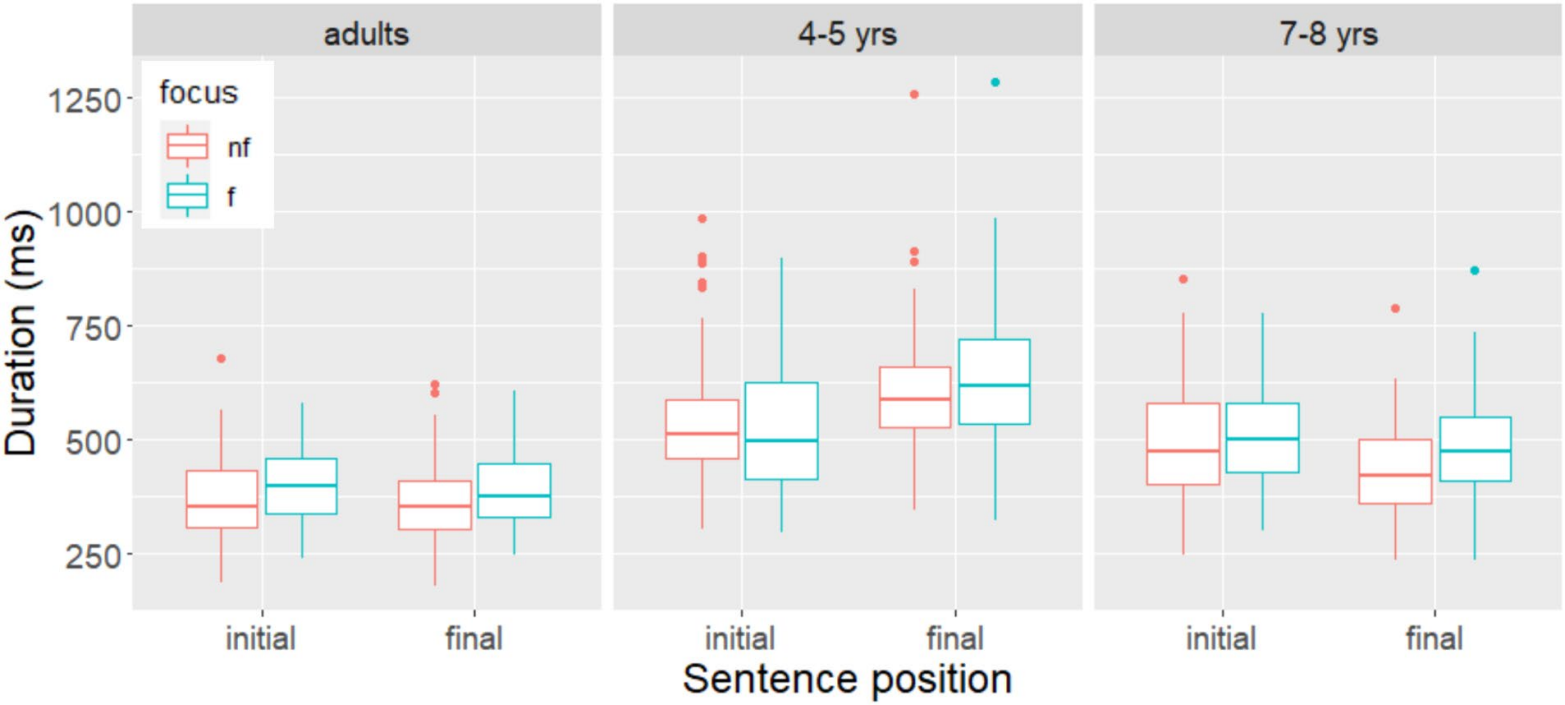
Duration on target nouns in non-focus and narrow focus, by group and sentence position. Non-focus is represented as “nf” (red); narrow focus as “f” (green).

For **duration**, the model with the best fit showed only a significant main effect of *Focus* (β = 30.322, SE = 6.072, t = 4.993, *p* < 0.001). As shown in Figure 2, the nouns were produced with a longer duration in the narrow focus condition than in the non-focus condition, regardless of sentence position.

**Figure 3:**
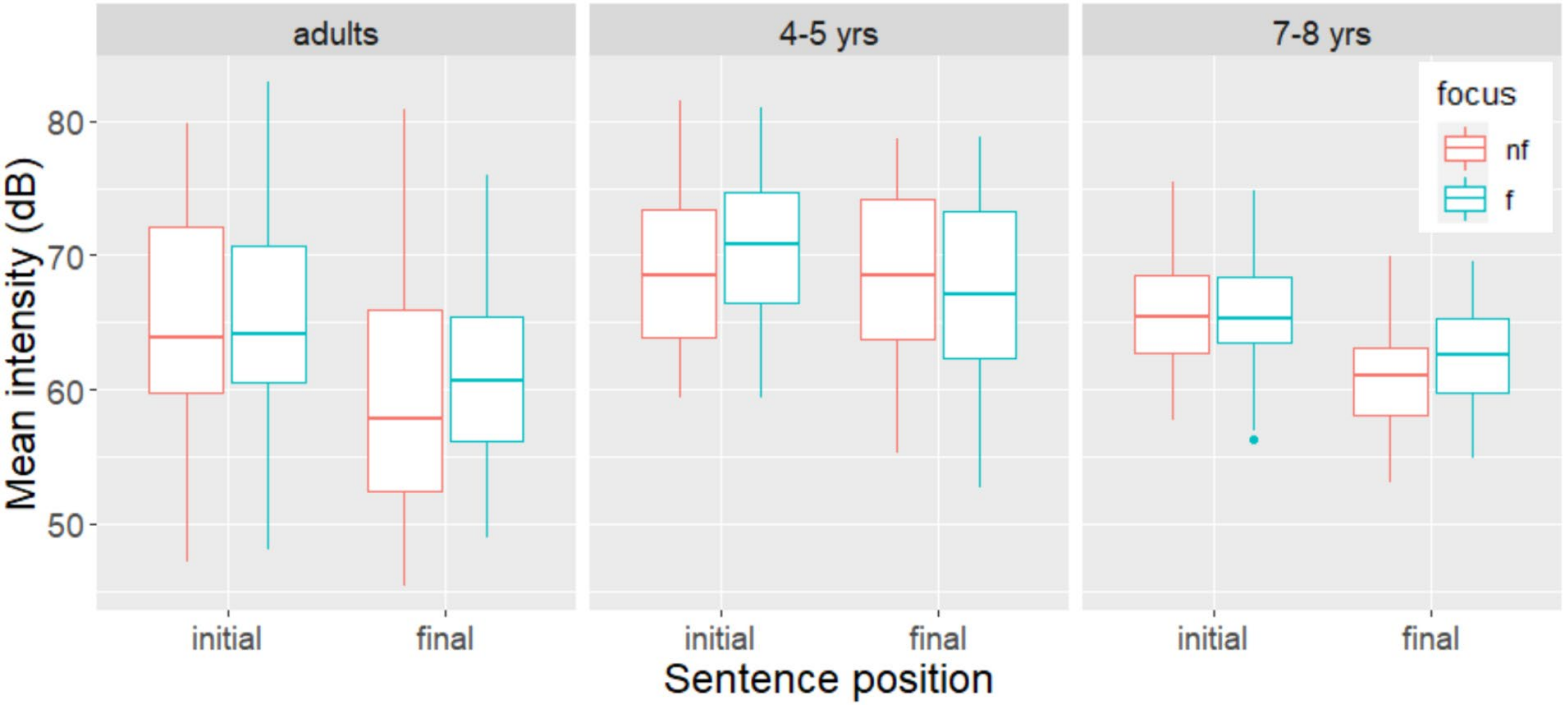
Mean intensity on target nouns in non-focus and narrow focus, by group and sentence position. Non-focus is represented as “nf” (red); narrow focus as “f” (green).

For **mean intensity**, the model with the best fit was the one that included both fixed factors and their interaction, revealing a significant interaction between *Focus* and *Position* (β = 1.407, SE = 0.485, *t* = 2.89, *p* < 0.004). Subsequent analysis showed that the main effect of *Focus* was only significant in final position (β = 1.95, SE = 0.359, *t* = 5.42, *p* < 0.001). Overall, the nouns were produced with a greater mean intensity in sentence-initial position than in sentence-final position; they were produced with a significantly higher mean intensity in the focused condition than in the non-focus condition only in sentence-final position, as shown in Figure 3.

**Figure 4:**
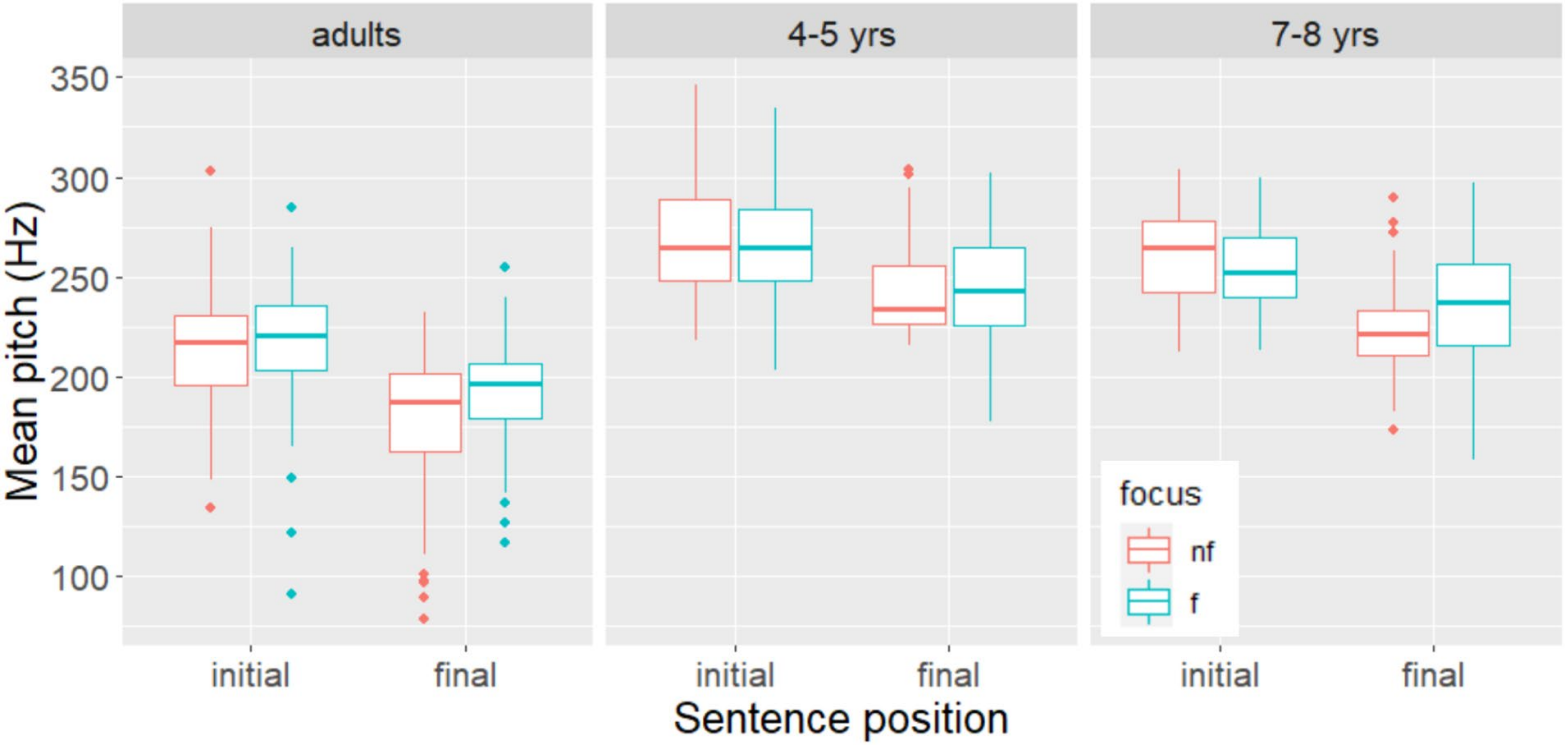
Mean pitch on target nouns in in non-focus and narrow focus, by group and sentence position. Non-focus is represented as “nf” (red); narrow focus as “f” (green).

Regarding **mean pitch**, the model with the best fit also showed a significant interaction between the fixed factors *Focus* and *Position* (β = 12.462, SE = 4.983, *t* = 2.501, *p* < 0.05). Subsequent analysis revealed that the main effect of *Focus* was only significant in sentence-final position (β = −15.772, SE = 3.748, *t* = −4.208, *p* < 0.001). That is, the nouns were produced with a significantly higher mean pitch in the focus condition than in the non-focus condition only sentence-finally, as can be seen in Figure 4.

Finally, the model with the best fit only showed a significant main effect of P*osition* for **pitch range** (β = 10.166, SE = 4.011, *t* = 2.535, *p* < 0.05).

To sum up, the adults made use of prosodic cues such as duration, mean intensity and mean pitch to signal narrow focus in sentence-final position, and only duration in sentence-initial position.

#### 5.1.2 4- to 5-year-old French-speaking children

The model with the best fit retained a significant main effect of *Position* for the outcome variables **duration** (β = 89.47, SE = 33.64, *t* = 2.65, *p* < 0.02), **mean intensity** (β = −2.341, SE = 0.55, *t* = −4.25, *p* < 0.001), and **mean pitch** (β = 26.042, SE = 2.158, *t* = 12.069, *p* < 0.001). Thus, the nouns in sentence-initial position were produced differently from those in sentence-final position. Regardless of whether they were focused or not, they had a shorter duration, a higher intensity, and a higher mean pitch in sentence-initial position (Figures 2–4). The best-fit model for the outcome variable **pitch range** contained only random factors. There was no effects of *focus* or *position*.

#### 5.1.3 7- to 8-year-old French-speaking children

The model with the best fit for the outcome variables **duration** retained a significant interaction between the fixed factors *Focus* and *Position* (β = 42.22, SE = 16.29, *t* = 2.59, *p* < 0.001). Subsequent analysis showed that the main effect of *Focus* was only statistically significant in sentence-final position (β = 54.08, SE = 10.25, *t* = −5.274, *p* < 0.001). Thus, the nouns were produced with a longer duration in the narrow focus condition than in the non-focus condition only sentence-finally, as shown in Figure 2.

The model with the best fit for the outcome variable **mean intensity** included a significant interaction between the fixed factors *Focus* and *Position* (β = 1.816, SE = 0.54, *t* = 3.32, *p* < 0.001). Subsequent analysis showed that the main effect of *Focus* was only significant in sentence-final position (β = 1.85, SE = 0.4125, *t* = 4.5, *p* < 0.001). Thus, the nouns were produced with similar intensity in the focus and non-focus conditions sentence-initially, but the nouns were produced with higher intensity in the focus condition than in the non-focus condition sentence-finally, as shown in Figure 3.

The model with the best fit for the outcome variable **mean pitch** only retained a marginally significant main effect of focus (β = −3.467, SE = 1.822, *t* = −1.903, *p* = 0.058). The nouns tended to be produced with a higher mean pitch when focused than when not focused, regardless of sentence position, as shown in Figure 4.

The model with the best fit for the outcome variable **pitch range** only showed a main effect of position (β = −19.373, SE = 4.563, *t* = −4.245, *p* < 0.001).

Taken together, these results show the 7- to 8-year-olds produced the sentence-final nouns with a longer duration and a higher intensity in the focus condition than in the non-focus condition in sentence-final position. Further, they tended to produce the nouns with a higher mean pitch in the focus condition than in the non-focus condition regardless of sentence position.

### 5.2 The effect of focus type: contrastive focus vs. narrow focus^5^

For adults, the model with the best fit for the outcome variable **duration** showed a main effect of *FocusType* (β = 23.06, SE = 7.54, t3.058, *p* < 0.005), such that the object nouns were produced with a shorter duration in contrastive focus than in narrow focus. No main effect of *FocusType* was found for the other outcome variables in the adult data.

No significant result appeared in the case of the 4- to 5-year-olds and the 7- to 8-year-olds, revealing that these participants did not use the prosodic cues investigated to distinguish between contrastive and narrow focus.

In sum, the findings indicate that the adults implemented only a decrease in duration to distinguish contrastive focus from narrow focus. The 4- to 5-year-olds and 7- to 8-year-oldfs did not differentiate these two focus types through prosodic cues.

## 6 Discussion and potential limitations

The findings on the French-speaking adults showed that duration, mean intensity and mean pitch were used to distinguish narrow focus from non-focus, similar to findings reported in previous studies on French. However, the results indicate an asymmetry in its realization between sentence-initial and final positions. While three prosodic parameters were used in sentence-final position, only duration was used to mark focus in sentence-initial position. This finding is in line with the observation that there are other non-prosodic strategies preferred by French-speakers for marking focus in sentence-initial position. Furthermore, the adults in our study produced the target words (i.e., the object nouns) with a shorter duration in the contrastive focus condition than in the narrow focus condition. This use of duration has not been reported before for French. It may be related to pragmatic reasons such as the intention to minimise potential negative connotation of correcting someone unfamiliar but of a similar age to oneself in the current set-up.

For the 4- to 5-year-olds, the current results have established that at this age, children do not implement focus through prosodic cues, in line with Hypothesis I. However, they were successful in manipulating these cues to distinguish sentence positions: similar to what is observed in adults from prior literature, 4- to 5-year-olds produced sentence-initial nouns with a higher mean intensity and a higher mean pitch than sentence-final nouns. Contrasting from adults, they additionally implemented the parameter of duration but were unsuccessful in using pitch range for this purpose. This suggests that although 4- to 5-year-olds are unable to signal focus through prosodic cues, they may already use some of these cues for other purposes. However, as the sentence-initial nouns were segmentally different from the sentence-final nouns, a direct comparison in their duration, mean intensity and mean pitch is not possible. Future research compared the same nouns in different sentence positions is needed to validate this speculation.

In the 7- to 8-year-olds, the findings indicate that, at this stage, children have developed some ability to use prosody for focus marking purposes, supporting Hypothesis I. Although they did not use prosody to distinguish focus types, i.e., contrastive focus and narrow focus, they used an increase in duration and mean intensity and to a lesser degree, an increase in mean pitch to differentiate narrow focus from non-focus in sentence-final position, similar to the French-speaking adults in our study. Furthermore, the 7- to 8-year-olds appeared to use an increase in mean pitch for this purpose in sentence-initial position, unlike the French-speaking adults who used duration for this purpose. These results indicate faster acquisition of prosodic focus marking in subjects than in objects, supporting Hypothesis II. As has become clear from section 2, subject-focus is preferably marked via cleft structures in French. There is evidence that children acquire such structures in focus contexts very early on, i.e., around the age of 2 years (Labelle, 1990; Belletti, 2005; De Cat, 2007; Soares-Jesel and Lobo, 2019; Lahousse and Jourdain 2024). Thus, the lower incidence of canonical Subject-Verb-Object sentences in subject-focus contexts could be a factor contributing to the later acquisition of prosodic focus marking in subjects.

We conclude our discussion by raising potential limitations of this study and proposing opening avenues for future research. One potential limitation relates to the observed difficulty some 4- to 5-year-old children had with certain words. More specifically, these children audibly struggled with pronouncing “docteur” (*doctor*) or “bâtit” (*built*). In cases where the error was on the noun, the trial was omitted from the analyses. However, since the verb was not included in the analyses, errors on these trials were maintained (given that there was no additional issue). Although this may seem unproblematic for the relatively few trials concerned, it is possible that if several children audibly struggled with these items, other children may have also had difficulties. A second point of attention is related to the fact that we have examined prosodic focus marking in children speaking the standard variety of French spoken in France. Due to prosodic differences between regional varieties of French (Obin et al., 2012), it can be insightful to replicate our study in children speaking prosodically distinct regional varieties of French. Finally, it remains to be investigated at what age exactly children acquire fully adult-like abilities in prosodic focus marking. Future research is needed to examine prosodic focus marking in older children, such as 10- to 11-year-olds based on previous studies on children acquiring other languages.

## 7 Conclusion

This study makes a new empirical contribution by providing insights into the development in the use of prosody for marking focus in French-speaking children. Our findings corroborate past literature on French-speaking adults on their use of prosody in focus marking in the absence of syntactic means and on French-speaking 4- to 5-year-olds on their lack of use of prosody in focus marking, showing differences between French-speaking children and children acquiring a language relying on prosody or both prosody and syntax to a similar degree for focus marking. Further, it provides first evidence that 7- to 8-year-olds use prosody to mark focus in sentence-final position (objects) in a more adult-like manner than in sentence-initial position (subjects), arguably caused by a more dominant role of syntactic means in marking focus on sentence-initial subject in French. Together, our study sheds new light on the influence of relative importance of prosodic and non-prosodic means on the acquisition of prosodic focus marking. Specifically, it shows that syntax-dominance can not only influence the route of acquisition (Chen, 2018) but also the rate of acquisition of prosodic focus marking.

## Data availability statement

The raw data supporting the conclusions of this article will be made available by the authors, without undue reservation.

## Ethics statement

Ethical review and approval was not required for the study on human participants in accordance with the local legislation and institutional requirements at the time of testing. The participants or the legal guardians provided their written informed consent to participate in this study.

## Author contributions

ED: Conceptualization, Funding acquisition, Investigation, Writing – original draft, Writing – review & editing. LL: Data curation, Formal analysis, Writing – original draft. AC: Conceptualization, Data curation, Formal analysis, Funding acquisition, Methodology, Project administration, Supervision, Visualization, Writing – original draft, Writing – review & editing.

## Footnotes

^1^Although the more natural way to respond to an open question is with a single constituent answer, sentences using the canonical order Subject-Verb-Object are commonly found in French, even in the spontaneous speech of 4-to-8-year-old children.

^2^The problematic NOCF item was removed from analysis due to a mismatch between the preposition used in the question and the preposition that participants produced in the answer. The question asked by the experimenter was of the form ‘Est-ce que les jumelles jouent *avec une* pierre? ‘*Are the twins playing with a stone?’*, which included the preposition ‘avec/with’. However, most of the answers given were produced with another preposition ‘au/at’, even though the robot answer was of the form ‘Les jumelles jouent *avec un* ballon *The twins play with a ball.*’

^3^Because the distance between the mouth of the participants and the microphone was not strictly controlled for adults and children, the mean intensity results reported here should be interpreted with caution.

^4^Our subsequent exploratory mixed-effects logistic regression modeling with *Group* (4–5 years, 7–8 years, or adults) has revealed a significant interaction of *Group x Focus x Position* in the model with the best fit for duration (*p* < 0.05), mean intensity (*p* < 0.05), and mean pitch (*p* < 0.05), but not for pitch range. These results suggest that differences observed between age groups in the use of pitch range need to be taken with caution.

^5^Our subsequent exploratory mixed-effects logistic regression modeling with *Group* (4–5 years, 7–8 years, or adults) has revealed a significant interaction of *Group x FocusType* in the model with the best fit for mean pitch (*p* < 0.001), but not for duration, mean intensity and pitch range. These results suggest that differences observed between age groups in the use of duration, mean intensity and pitch range need to be taken with caution.
