# Supplementary material for: The development of prosodic focus marking in French
Source: Front Psychol. 2024 Jul 25;15:1360308. doi: 10.3389/fpsyg.2024.1360308 (PMC11345614; doi:10.3389/fpsyg.2024.1360308)
Supplement: Supplementary file 1 [file Data_Sheet_1.pdf]

Supplementary Material

1.2 Supplementary Data set

List of all stimuli used in experimental task (8 per condition)

**Condition**	**Question asked by the experimenter**	**Expected SVO answer**
Contrastive Focus Object	Est-ce que la souris mange du fromage?	La souris mange un bonbon.
	Est-ce que le boucher découpe des saucisses?	Le boucher découpe un jambon.
	Est-ce que le canard mange une fraise?	Le canard mange un marron.
	Est-ce que le mouton mange du baguette?	Le mouton mange des raisins.
	Est-ce que le papie lit un livre?	Le papie lit un journal.
	Est-ce que le pirate vole un bâteau?	Le pirate vole un vélo.
	Est-ce que le renard attaque un canard?	Le renard attaque une maison.
	Est-ce que les jumelles bottent une pierre?	Les jumelles joue au ballon.
Narrow Focus Object	Que nettoie la mamie?	La mamie nettoie la maison.
	Que mange le bébé?	Le bébé mange un bonbon.
	Qu'achète le docteur?	Le docteur achète un journal.
	Que peint le garçon?	Le garçon peint un ballon.
	Que mange le lapin?	Le lapin mange des raisins.
	Que mange le lézard?	Le lézard mange un marron.
	Que répare le soldat?	Le soldat répare un vélo.
	Que vole le voleur?	Le voleur vole un jambon.
Narrow Focus Subject	Qui joue au ballon?	Un bébé joue au ballon.
	Qui lit le journal?	Un docteur lit le journal.
	Qui mange le bonbon?	Un garçon mange le bonbon.

	Qui mange le maron?	Un lapin mange le marron.
	Qui mange les raisins?	Un lézard mange les raisins.
	Qui bâtit la maison?	Un soldat bâtit la maison.
	Qui fait du vélo?	Un voleur fait du vélo.
	Qui découpe le jambon?	Une mamie découpe le jambon.
