# Supplementary material for: The development of prosodic focus marking in French
Source: Front Psychol. 2024 Jul 25;15:1360308. doi: 10.3389/fpsyg.2024.1360308 (PMC11345614; doi:10.3389/fpsyg.2024.1360308)
Supplement: Supplementary file 2 [file Table_1.pdf]

Supplementary Material

1.1 Supplementary Tables

Supplementary Table 1. *Usable trials (in bold) out of the total number of trials collected for each condition.*

Group *Focus condition*	*NSF*	*NOF*	*NOCF*
**Children group 1** (4-5 yrs, n = 10)	**52**/*80*	**58**/*80*	**41**/*70*
**Children group 2** (7-8 yrs, n = 15)	**86**/*120*	**91**/*120*	**54**/*105*
**Adults** (17-44 yrs, n=11)	**86**/*88*	**88**/*88*	**74**/*77*
